# The noise spectrum influence on Noise-Induced Hearing Loss prevalence in workers

**DOI:** 10.1016/S1808-8694(15)30646-7

**Published:** 2015-10-19

**Authors:** Marlene Escher Boger, Anadergh Barbosa-Branco, Áurea Canha Ottoni

**Affiliations:** 1 MSc in Health Sciences, Speech and Hearing Therapist; 2 PhD in Occupational Health. Professor - Universidade de Brasília - UNB; 3 PhD Student in Health Sciences. Speech and Hearing Therapist. Laboratório de Saúde do Trabalhador - Departamento de Saúde Coletiva - Faculdade de Saúde - Universidade de Brasília

**Keywords:** audiometry, occupational exposure, hearing loss, noise

## Abstract

Noise Induced Hearing Loss (NIHL) is an insidious and cumulative disease that worsens over the years with work-related noise exposure.

**Aim:**

To evaluate the noise spectrum influence on NIHL prevalence in workers.

**Materials and Methods:**

This a cross-sectional historical cohort carried out in steel mills, lumber mills and marble shops, with noise levels above 85dB, in which we evaluated the auditory thresholds for frequencies from 250Hz to 8,000Hz. To evaluate the work environment, we observed the entire setting, aiming at checking sound intensities in an eighth frequency filter.

**Results:**

We carried out 192 hearing threshold evaluations after an occupational anamnesis. Concerning NIHL, we noticed that 49% of the audiometry results presented hearing deterioration in the acute frequencies. We studied the mean values and standard deviations for frequencies over 3,000Hz, in all workers, and we observed that the highest average values were in the frequency of 6,000Hz. We did not notice any association among frequency bands carrying intense noise levels and the hearing damage frequency.

**Conclusion:**

Noise intensity seems to be the main risk factor for loss hearing, regardless of frequency range.

## INTRODUCTION

Noise Induced Hearing Loss (NIHL) has many names, such as “Hearing Loss by Noise Exposure”, “Occupational Hearing Loss”, “Professional Hearing Loss”, “Professional Deafness”, “Occupational Dysacousia”, “Occupational Noise Induced Hearing Loss” and “Sensorineural Hearing Loss by Continuous Exposure to High Levels of Occupational Hearing Loss”; however, they are all part of a profession-related disease, characterized by a gradual reduction in hearing acuity because of continuous exposure to intense levels of sound pressure, causing damage to outer and inner hair cells of the Organ of Corti^1^, which according to research is one of the most prevalent diseases today.^2^

According to the National Institute of Occupational Safety and Health^3^, noise is one of the major health problems in the USA today, and that there are approximately 30 million workers exposed to hearing harmful levels of noise in their work environment.

It is estimated that there are approximately nine million American workers with hearing loss caused by occupational exposure to noise. In developing countries, the situation is usually more severe, since it is common for workers to be exposed to intense noise levels in their work places, besides low compliance with the measures used for collective and individual protection.^4^

There is a consensus in the literature that the duration of noise exposure is associated with NIHL onset. In the study carried out with bus drivers in Campinas, they found a positive association between NIHL and accumulated duration of noise exposure.^5^

According to the World Health Organization, the excessive exposure to noise can cause other health problems, such as auditory stress under exposures of 55 dB; physical reaction such as blood pressure raise, heart rhythm and muscle contractions; the increase in adrenaline production and other hormones; irritability; stress; insomnia and anxiety.^6^

Epidemiologic studies reveal that occupational hearing problems affect more frequently workers of the following occupations: steel workers, mechanics, graph, textile, chemist/petrochemist, transportation and those in the food and beverage industry, industries with high noise levels.^7^

In the metropolitan region of Salvador, Bahia, a prevalence study was carried out based on the audiometric data of workers from 44 plants in nine different industries. Such study revealed that 45.9% of the population studied had hearing loss. As far as NIHL is concerned, the prevalence seen was of 35.7%. The highest prevalence rates were found in the editorial/graph (58.7%), mechanic (51.7%), beverages (45.9%), chemist/petrochemist (42.3%), metallurgic (35.8%), steelworker (33.5%), transportation (29.3%), food (28.0%) and textile (23.4%) industries, corroborating with the literature.^8^

A study carried out with workers exposed to noise in marble mills at the Federal District found a high prevalence of hearing damage as well (48.0%) and association between exposure duration and NIHL.^9^ A similar study carried out with 187 workers from the metallurgical industry, in the city of Goiania, reveals that 22.0% of the workers assessed presented hearing damages suggestive of NIHL.^10^ These differences may be, in part, stemming from the characteristics of the samples studied, considering important factors in the process such as exposure duration, compliance with wearing protection equipment, etc.

Considering the problem relevance, this study intends to carefully assess the work environment where the worker is exposed, the noise spectrum and the influence it has on the workers' hearing.

## METHOD

This is a cross-sectional epidemiological study of historical cohort, carried out in different industries with noise levels above 85 dB, in which we assessed their hearing thresholds and through an occupational anamnesis we identified occupational data, life styles and issues associated with morbidity.

The population under study was selected by means of a convenience sample, made up only by those companies which agreed to participate in the study. The companies are geographically restricted to the Federal District area, and the industries assessed were: marble mills, timber mills and metallurgic mills.

We assessed workers between the ages of 18 and 65 years, males and with a minimum of 1 (one) year on the job. Workers from these industries with occupational noise below 85 dB, workers with a past history of hearing alterations of air conduction or mixed type, and workers with a history of acoustic trauma.

The audiologic assessment was preceded by an ear canal inspection and acoustic rest of 14 hours. Such assessment was carried out in order to check for the minimum response level for frequencies 250 Hz, 500 Hz, 1,000 Hz, 2,000 Hz, 3,000 Hz, 4,000 Hz, 6,000 Hz and 8,000 Hz. The bone pathway was tested only when the hearing thresholds exceeded 25 dB HL, in the frequencies of 500 Hz, 1,000 Hz, 2,000 Hz, 3,000 Hz and 4,000 Hz.

In order to classify hearing thresholds, we used the audiometric alteration criteria that classifies in accordance with the mean values of the 500, 1,000 and 2,000 Hz frequencies. The audiometries with scores suggesting Noise Induced Hearing Loss were the ones with normal reports and a notch in the frequencies of 3,000 Hz, 4,000 Hz, 6,000 Hz and or 8,000 Hz or characterizing a sensorineural curve with drops in the high frequencies and descending curve with a notch shape.

Each worker underwent an audiometric exam. The equipment used in the audiologic evaluation were: a Welch Allyn otoscope with WA accessories; Interacoustics clinical audiometer, AC 40 model with two channels and frequency range of 125 Hz to 16,000 HZ; Redusom sound-treated booth, model RO-80 Std. All the equipment was calibrated according with Standards ISO 389/64 and ANSI S3.6/69 duly registered. The audiometric booth has internal noise patterns within levels accepted by law, about 30 dB HL, as per ANSI S3.1 from 1991.

The environmental evaluation was carried out at two moments. In the first one, we noticed the sound intensity distribution in an octave frequency filter, using an equipment that measures sound pressure levels (SPL). This assessment was carried out in central points of the plant environment. To do that, we used one sound pressure level measurement device, of the SIP 95 brand from the 01dB manufacturer, with a built-in frequency analyzer, duly calibrated. Data collection time in each plant was of approximately 30 minutes.

The equipment equalizing circuits has the option of choice for curves A, B, C and D; two time constants, which were: slow and fast.

The A-Weighed curve damps the low sounds, providing greater gain for the 2 to 5 kHz frequency band, and then it goes back to damping the high-pitched sounds. This is exactly the hearing aid sensitivity curve. The C-weighed curve is linear and is incorporated to the measuring devices when there is the need to measure the entire room sound level, or to assess the presence of low frequency sounds. For this study we used the linear-weighed curve, since the objective of this analysis is to measure the sound of the entire room with precision.

In a second moment, we analyzed the individuals' exposure to noise during their work hours. The device used for this analysis is the dosimeter, capable of measuring the worker's true exposure, because the latter continuously follow up on all the noises that reach the individual during their work hours, providing the mean value. For such a measurement, we used the 01dB dosimeter, made in Brazil, which the worker during 4 (four) hours of work. For such analyses, we selected 2(two) workers from each plant. Both equipment pieces were placed in such a way that the microphones were at approximately 20 cm for the worker's ear.

The daily exposure level was calculated according to the following expression:

L
exp
=
L
eq
+
10
×
log
(
T
exp
T
0
)

For these calculations, we used the mean time of daily exposure (Texp) of 9 hours (according to information obtained from the workers) for 8 hours of work (T0), Leq represents the equivalent level of sound pressure, according to NBR 10.151 standard.

In order to calculate the percentage dose of noise absorbed daily, the following expression was used:

D
=
T
exp
8
×
2
(
L
c
r
i
t
−
L
ep
)
/
q
×
100
%

For the exchange factor q, the value of 5 was used and Lcrit of 85 dB(A), according with the NR 15 standard.

The estimate of the maximum time of Tt tolerated exposure by the workers of the metallurgical, timber and marble mill plants, resulted from expression 3.

T
t
=
8
×
2
(
L
c
r
i
t
−
L
eqt
)
/
q

The audiologic evaluation information data was stored in Microsoft Excel, thus performing the statistical analysis (mean, median, standard deviation and prevalence coefficient). In order to analyze the results obtained, we used the t Student statistical test (in order to evaluate the mean and standard deviation) and the Fisher Exact Test (in order to assess the prevalences), both with a 95% level of significance.

This paper was submitted to the Ethics in Research Committee - Faculdade de Ciências da Saúde da UnB, protocol # 048/2004.

## RESULTS

We carried out 192 evaluations of hearing thresholds (audiometry), preceded by an occupational patient interview. Of these, 91 tests in 2 metallurgical plants, 54 in 3 timber mills and 47 in 5 marble mills.

The workers evaluated had mean age of 34.6 years (SD± 8.8); and 38.5% were 30 years old or younger. As far as gender is concerned, we only assessed male workers.

Data regarding individual protection equipment, collected prior to the audiometric test, can be observed on Table 1.

**Table 1:** Sample data collected during the patient interview according to the use of ear IPE and the industry, Distrito Federal, 2006-2007.

EAR IPE	METALLÚRGIC		TIMBER		MARBLE		ALL	
	Use	%	N	%	N	%	N	%
Yes	86	94,5	16	29,6	43	91,5	145	75,5
No	4	4,4	26	48,1	2	4,3	32	16,7
Rarely	1	1,1	12	22,2	2	4,3	15	7,8
TOTAL	91	100,0	54	100,0	47	100,0	192	100,0

In relation to the industries, this variable pointed out that the timber mills which are the ones that least used this equipment, 48.1% of the employees stated they did not use it and 29.6% said they rarely use it. Nonetheless, the metallurgical plants and the marble mills were the ones that most used IPE. (Table 1)

According to audiometric results, we have observed that 49.0% of the workers evaluated had a notch in the high frequencies, shown in the audiogram (3,000; 4,000; 6,000 and 8;000 Hz). Among others, 13.8% in the right ear, 23.4% in the left ear and 62.8% in both ears.

As far as the industry is concerned, the results point out that 53.8% of the workers from the metallurgical plants had an audiometric notch, followed by those from the timber mills, with 48.1%, and marble mills with 40.4%.

As we assess auditory damage laterality, associating it with the person's occupation, we noticed that in all the industries there was an audiogram notch in both ears, and the metallurgical plant individuals had equal prevalence in the right and left ears. As to the timber and marble mills, we observed a tendency towards laterality, since in these industries the left ear had high prevalence when compared to the right ear. We did not see statistically significant differences among the industries (p>0.05-Fisher Exact Test). (Table 2)

**Table 2:** Prevalence of a NIHL notch according to the industry and laterality, Distrito Federal, 2006-2007.

NIHL NOTCH	METALLÚRGIC	TIMBER	MARBLE	ALL INDUSTRIES
	%	%	%	%
RE	18,4	7,7	10,5	13,8
LE	18,4	23,1	36,8	23,4
BE	63,3	69,2	52,6	62,8
TOTAL	100,0	100,0	100,0	100,0

Legend: RE right ear; LE left ear; BE Both ears

As we analyzed the workers in relation to the average of audiometric thresholds according to the frequencies starting at 3,000 Hz, we observed that the highest averages were found in the frequency of 6,000 Hz, characterizing the presence of a notch configuration. In the analysis of variation among the frequencies in each ear alone, we noticed that it was highly significant (p<0.0001-t Student test) in all the frequencies for both the right and the left ears.

As we studied the mean and standard deviation values of the audiogram thresholds of workers according with age range and the frequencies starting at 3,000 Hz it was possible to see that as age progresses, there is an increase in the degree of loss, and we can state that at an older age (> 50 years) the workers presented mild to moderate losses involving frequencies starting at 3,000 Hz. When we compared the auditory thresholds between the left and right ears, we noticed no statistically significant differences (p>0.05-t Student test), although the left ear had worse auditory thresholds in some frequencies.

In analyzing the mean values of audiometric thresholds according to frequencies starting at 3,000 Hz and the time of exposure, we observed that the longer the time of exposure, the greater the degree of hearing loss, with significance (p< 0.05-t Student test) in all the frequencies, when compared among themselves.

According to the environmental evaluation, it was possible to see that in each one of the industries studied, there was a result which was different among the environmental noise spectrums. We notice that for each frequency band, there is an equivalent level of sound pressure represented in decibel.

In the metallurgical plants, the 8,000 Hz frequency band is the one that represents the highest noise level (85.4 dB); therefore, we see that it prevails in the environment, and it is the one most noxious to workers. Nonetheless, we see that the differences among levels of sound intensity are similar among the other frequency bands. We notice that among the octaves that go from 250 all the way to 8,000 Hz, the intensity difference is low, showing that the sound energy in this industry is almost uniformly distributed among these frequency bands. (Table 3)

**Table 3:** Environmental noise spectrum broken down by industry, Distrito Federal, 2006-2007.

Octave band	INDUSTRY		
	METALLÚRGIC	TIMBER	MARBLE
	Leq (dB)	Leq (dB)	Leq (dB)
31.5Hz	71,5	62,4	64,2
63Hz	71,7	60,1	63,8
125Hz	76,6	65,8	63,1
250Hz	82,6	72,2	66,6
500Hz	81,8	73,9	71,0
1kHz	81,2	78,6	73,2
2kHz	81,0	80,5	74,9
4kHz	82,2	73,7	79,3
8kHz	85,4	71,0	71,7
Leq global	91,0	84,3	82,5

L_eq_ Sound pressure equivalent level.

As far as timber mills are concerned, this analysis reveals that the 2,000 Hz frequency band is the one which prevails in the environment, and this may very well be the one that causes the worst damage, since among the others, this is the one that has the highest sound pressure level (80.5 dB). We also see that, contrary to what is seen in the metallurgical plant, the distribution of sound energy does not remain equivalent among the many frequency bands, it is possible to see that the octaves 1,000 Hz and 2,000 Hz have the highest intensity levels (Table 3).

We see that marble mills have less noxious levels to the health of workers, the spectral analysis reveals that the highest noise level was of 79.3 dB in the 4,000 Hz frequency band, which prevails in the environment. Global intensity levels in the environment in each one of the industries we studied can be seen on Table 3. These results come from the assessment carried out with the sound pressure level measuring device.

Based on the noise dose assessment (dosimeter), we see that the results presented different sound pressure levels when compared to those on Table 3. This analysis revealed that in each one of the industries assessed, the noise level was higher than dB, just like daily exposure levels, and in three of the industries studied, timber was the one with the highest sound pressure levels, with a dose corresponding to 2,924.1%. In this case, the tolerance time for the worker to stay in the environment without wearing proper protection is of 18.4 minutes. In relation to a possibly more harmful place to work, we see that the timber industry comes in first place, followed by metallurgical and marble, respectively. (Table 4)

**Table 4:** Sound pressure equivalent level, daily exposure level, dose and time of maximum exposure tolerated according to the industry, 2006-2007.

INDUSTRY	Leq (dB)	Lexp (dB)	Dose (%)	Time tolerated (min)
Metallurgic	103.3	103.8	1422.0	37.9
Timber	108.5	109.0	2924.1	18.4
Marble	104.5	105.0	1679.4	32.1

L_eq_ Sound pressure equivalent level; Lexp Daily exposure level.

Comparing audiometric thresholds with results from spectral analyses and the different industries, we did not observe any association among the frequency octave bands with the higher levels of noise and the auditory damage frequency. We notice that frequencies with the highest thresholds do not coincide with the frequency bands which intensity levels reach higher values. We also observe that, despite the difference between noise spectrum in these three industries, the mean values of audiometric thresholds are similar. (Table 5)

**Table 5:** Comparison among audiometric thresholds and the spectral analysis results according to the frequency octave bands and the industries, Distrito Federal, 2006-2007.

OCTAVE BAND	INDUSTRIES								
	METALLÚRGIC			TIMBER			MARBLE		
	Leq (dB)	RE	LE	Leq (dB)	RE	LE	Leq (dB)	RE	LE
31.5Hz	71,5	–	–	62,4	–	–	64,2	–	–
63Hz	71,7	–	–	60,1	–	–	63,8	–	–
125Hz	76,6	–	–	65,8	–	–	63,1	–	–
250Hz	82,6	15,5	15,3	72,2	14,4	13,3	66,6	16,1	15,8
500Hz	81,8	12,4	12,4	73,9	12,1	11,8	71,0	13,0	12,3
1kHz	81,2	11,2	11,3	78,6	10,4	10,2	73,2	9,5	9,6
2kHz	81,0	10,3	11,5	80,5	12,0	11,5	74,9	9,5	10,8
4kHz	82,2	21,5	20,6	73,7	20,3	22,8	79,3	18,4	19,1
8kHz	85,4	17,8	16,9	71,0	15,6	16,6	71,7	15,4	17,1
Leq global	91,0	–	–	84,3	–	–	82,5	–	–

L_eq_ Sound pressure equivalent level; RE right ear; LE left ear.

## DISCUSSION

Workers' hearing health has received growing attention in the recent decades, since the problems found are not limited only to hearing loss. Many studies stress the extra-auditory effects of noise, occlusion effects caused by protective phones, temporary threshold alteration, tinnitus and others.^6^

We see that among the three industries analyzed, timber mills were the ones showing the lowest compliance with ear protection (IPE), suggesting less attention paid to issues of prevention, corroborating with the high prevalence in those who state they do not wear protection. Nonetheless, the metallurgical and marble mill plants, which were found to be more zealous as far as the use of protection equipment is concerned, also pointed to an alarming number of hearing damage, reinforcing the results of many studies which prove that the choice of equipment must be equivalent to its real need, since for each type of noise exposure, a specific type of ear protection is recommended, which can vary among the types: cup, plugs, and others.^11^, ^12^, ^13^

We stress that the ear IPE used by the workers hereby discussed, suggest little effectiveness, since in all the industries evaluated, there is a relevant prevalence of workers who developed some kind of hearing damage in at least one of the ears, and in at least one frequency.

The issue of hearing loss laterality has been discussed by many authors who state that NIHL is usually bilateral.1 Such event was observed in this study, since the highest prevalence of NIHL notch happened in both ears. However, the left ear proved to be slightly more prone to it than the right ear. As far as a possible asymmetry, some authors report that the left year is more vulnerable to noise-induced damage, however, there was no evidence for such statement.^14^ Another study considered that male adult hearing is about 4 dBHL lower on the left ear, when compared to the right one.^15^ This has also been observed in clinical practice, it is possible to perceive that during audiometry we have a better right ear response when compared to the left ear. The possible physiological mechanisms responsible for this difference seem to be unknown.

The high prevalence of notches in NIHL in the left ears that we noticed in timber and marble workers may, at least partially, be explained by the fact that, contrary to what was observed in metallurgical plants, most marble and timber plants are located in buildings which are closed on one side and open on the other, probably because of the excess dust in these environments, and with this, the physical organization of the production areas can influence and cause a greater involvement of one ear in relation to the other; nonetheless, further and more specific studies should be carried out in order to confirm this causality relationship.^9^ The physical structure of these environments can also justify the global equivalent intensity level found in each industry, resulting from the sound pressure level measuring device, since in this analysis the metallurgical plants had the highest result. Scholars advocate that the sound reverberation time in a closed area is higher than that in an open field.^16^

As we analyze the mean obtained in the frequencies of 3,000 to 8,000Hz of the entire sample, we observed that in 6,000Hz we find the mean values above 25 dB, in other words, the auditory damage seems to affect mostly these frequencies. Recent studies which used only conventional audiometry, point out that 6,000Hz was the first frequency to be affected because of exposure to occupational noise.^3^ In another study carried out with the goal of investigating the hearing of people exposed to occupational noise in conventional and high frequencies, they assessed individuals exposed to noises above 90 dB SPL (study group) and 30 without exposure to noise and without a past of hearing problems (control group).^17^ The results found show that according to an increase in frequency, age and duration of noise exposure, the greatest acuity loss happened in the control group.^17^

One of the points that most calls our attention in this study is the result involving the two stages of the environmental analysis. We see that the data regarding sound pressure intensity in the environment is different, and based on the dose assessment, noise is more intense in all the industries. Such event may be justified by the way through which the data was collected, since the sound pressure level monitor, at the time of the assessment, was placed in a central point within the plant, capturing the entire environment sound. Now, regarding the evaluation carried out with the dosimeter, this time the equipment had been placed next to the worker's ear, whom remained in direct contact with the production machine, thus capturing higher levels of sound intensity. Scholars have revealed that there is a reduction in sound propagation by air, stressing that low frequency sounds are easier to convey by air when compared to high frequency sounds. Thus, the high pitch noise lose their power in a short distance, thus justifying the findings from this study.^16^

It is known that the Organ of Corti injury mechanism is based on the cochlear basal turn - the area responsible for the high frequency sounds^1^, regardless of the frequency spectrum of the harming noise, being considered damaging according with the noise intensity.^18^ This justifies the results from the noise spectrum found in the industries we studied, since for each industry, the frequency band considered more damaging is the one which intensity values, represented in dB, reach the highest peaks. Such involvement may be associated, in part, to the type of raw material used in each industry, since it is believed that timber cutting, for instance, produces an intense noise in a lower frequency when compared to metal or marble, this is due to the particularities of the physical properties which make up each material. We also stress that, even with different spectral results, the three industries studied have a similar hearing damage potential both in frequency as in level of the hearing loss.

The lack of association between the frequency bands with intense noise levels and the hearing damage frequency found in the present investigation also justify the findings from a research paper which discussed the prevalence of NIHL in bus drivers.^5^ It is believed that the noise transmitted by bus engines is in a lower frequency when compared to industrial noises, even then, we see that bus drives, despite being exposed to a different noise spectrum, have hearing losses which are similar to those from industry workers, primarily affecting the high frequencies of the audiogram.

We also stress that the lack of association among the frequency bands of the noise spectrum and the hearing loss frequency does not rule out the importance of this type of environmental analysis, since in order to obtain a thorough and efficient program for occupational noise control it is extremely important to know the frequency bands that bear the most intense noise levels, in order to establish safety measures, thus facilitating the creation of projects to reduce sound levels.^16^

## CONCLUSION

The environmental analysis did not show association between the frequency bands and the intense noise levels and hearing loss frequency. Given that, despite the three industries having presented different noise spectrum results, the hearing loss pattern is similar among them. Thus, in this case it is possible to state that the noise intensity seems to be the major risk factor associated with the hearing loss. As to noise induced hearing losses (49.0%), we noticed that the frequency bearing the biggest notches is 6,000 Hz.
